# Development of a 3D-Printed Wistar Rat Model From Digital Imaging and Communications in Medicine (DICOM) Data for Medical Education and Research Applications

**DOI:** 10.7759/cureus.78012

**Published:** 2025-01-26

**Authors:** Todor G Bogdanov, Todor A Hikov, Zafer A Sabit, Radka Tafradzhiyska-Hadzhiolova, Daliya T Pencheva

**Affiliations:** 1 Medical Physics and Biophysics, Medical University - Sofia, Sofia, BGR; 2 Physiology and Pathophysiology, Medical University - Sofia, Sofia, BGR

**Keywords:** 3d anatomical models, education model, medical education, skeleton, three-dimensional (3d) printing, wistar rat

## Abstract

Three-dimensional (3D) printing has revolutionized medical education and research by enabling the creation of accurate anatomical models derived from imaging studies. This report details the development of a 3D-printed Wistar rat model, produced using DICOM (Digital Imaging and Communications in Medicine) data from a single radiological study. The model was designed to serve as an educational tool for medical and veterinary students and as a resource for researchers.

DICOM data from a single animal study was segmented and converted into a high-resolution 3D model using specialized software that converts the voxels from the slices into a waterfall volume with a uniform surface. The resulting STL file underwent optimization to ensure compatibility with common 3D printing technologies, such as fused deposition modeling (FDM) and stereolithography (SLA). The final printed model replicates the intricate anatomical details of the Wistar rat, providing a valuable aid for studying rodent anatomy and simulating procedures in preclinical research.

The STL file is freely accessible to researchers and educators, promoting the adoption of 3D-printed models in academic settings. Providing open access to this model, we aim to enable researchers, educators, and clinicians worldwide to utilize it for various purposes, including comparative anatomy studies, surgical training, and educational demonstrations. This approach significantly enhances the usability of our findings and fosters collaboration across disciplines, making the model a valuable resource for the scientific community. By integrating advanced imaging techniques with 3D printing, this study demonstrates a cost-effective and scalable approach to enhancing medical education and research capabilities.

## Introduction

In medical [1] and veterinary education [2], accessing and interacting with accurate anatomical models is crucial for fostering a deeper understanding of biological structures and systems. Traditional methods for producing anatomical models often rely on cadaveric specimens or commercially manufactured synthetic models. While these resources have been valuable, they come with inherent limitations. Cadavers are not always readily available, their use raises ethical and logistical concerns, and their preservation requires significant financial and institutional resources. Synthetic models, on the other hand, are often costly, lack customization options, and may not accurately represent specific anatomical variations. Furthermore, the effectiveness of such models in enhancing learning outcomes is closely linked to individual learning styles, as highlighted by a previous study, which demonstrated that learning styles significantly influence study duration and academic success [3]. This finding underscores the importance of creating accessible and customizable resources that cater to diverse educational needs.

The advent of three-dimensional (3D) printing technology has introduced a paradigm shift in how anatomical models can be developed and utilized [4,5]. By integrating imaging techniques, such as computed tomography (CT) or magnetic resonance imaging (MRI), with advanced 3D modeling software, researchers and educators can create highly detailed and anatomically precise models tailored to specific needs [6]. This innovation has significant implications for both educational and research settings, offering a cost-effective, scalable, and highly customizable solution for teaching and practice.

The Wistar rat is prominent in biomedical research [7] as a model organism for studying human diseases, testing pharmaceutical interventions, and advancing surgical techniques. The model can be used as a hardware model for playing out operational approaches or as a software model (shared with open access as a 3D file) for the purpose of adding it to simulation software for augmented and/or virtual reality. Its anatomical and physiological similarities to humans in certain aspects make it an ideal candidate for translational research. Since their creation in 1906, Wistar rats have been widely utilized due to their predictable biology, calm temperament, and adaptability to laboratory conditions [8]. Despite its widespread use in research, educational tools representing the anatomy of the Wistar rat are often limited to two-dimensional (2D) diagrams or digital models, which can lack the tactile and spatial benefits of physical 3D models. A detailed and accurate 3D-printed model of the Wistar rat has the potential to fill this gap, serving as a valuable resource for medical and veterinary students and researchers alike.

This report outlines the development of a 3D-printed Wistar rat model derived from DICOM imaging data. The workflow begins with image acquisition and segmentation, continues through 3D model reconstruction and optimization, and concludes with the creation of a printable STL file. The STL file is made freely available to educators, students, and researchers, providing a resource that can be easily accessed and produced using standard 3D printing technologies, such as fused deposition modeling (FDM) or stereolithography (SLA).

The objectives of this study are to provide a comprehensive methodology for converting DICOM imaging data into a 3D-printable model, to showcase the educational and research benefits of utilizing a physical 3D model of the Wistar rat, and to promote accessibility and adoption of 3D-printed anatomical models by sharing the STL file with the academic and research community. By bridging the gap between advanced imaging techniques and 3D printing technology, this study aims to enhance the educational experience of medical and veterinary students while offering a valuable tool for preclinical research. The integration of these technologies not only democratizes access to high-quality anatomical models but also paves the way for the broader adoption of 3D printing as a standard practice in education and research.

## Technical report

The imaging study, serving as the basis for the development of the 3D model, was performed using a 16-slice General Electric computed tomography (CT) scanner (General Electric Company (GE), Boston, MA), manufactured in 2010. The acquired dataset included 441 slices with a slice thickness of 0.6 mm, ensuring high spatial resolution and the ability to capture detailed anatomical structures. The slices were recorded in DICOM (Digital Imaging and Communications in Medicine) format, a widely used standard for medical imaging that guarantees compatibility with software for further processing.

The methodology employed in the creation of the model follows the detailed steps described in a prior publication [9]. During the processing of the DICOM data, a region of interest (ROI) was selected, limited to a range between 226 and 1785 Hounsfield units (HU). This range was chosen to precisely differentiate bone structures from soft tissues, ensuring high anatomical accuracy in the reconstructed model while completely eliminating the visualization of soft tissues in the imaging data. The exclusion of soft tissue visualization was further facilitated by the absence of contrast agents during the CT scan, which could otherwise introduce artifacts and false structures into the reconstructed model. This decision ensures a cleaner and more anatomically accurate representation.

The acquired data underwent several critical steps using modern software tools and 3D modeling technologies such as DICOM data processing using InVesalius 3 (Centro de Tecnologia da Informação Renato Archer, Campinas, Brazil), an open-source software specifically designed for generating three-dimensional models from medical images. The reconstruction process involved the following steps of segmentation of bone structures from soft tissues, focusing on the selected range of 226 to 1785 HU, manual refinement of the region of interest when necessary to ensure high anatomical precision, and exporting the segmented three-dimensional model in STL format for further processing.

**Figure 1:**
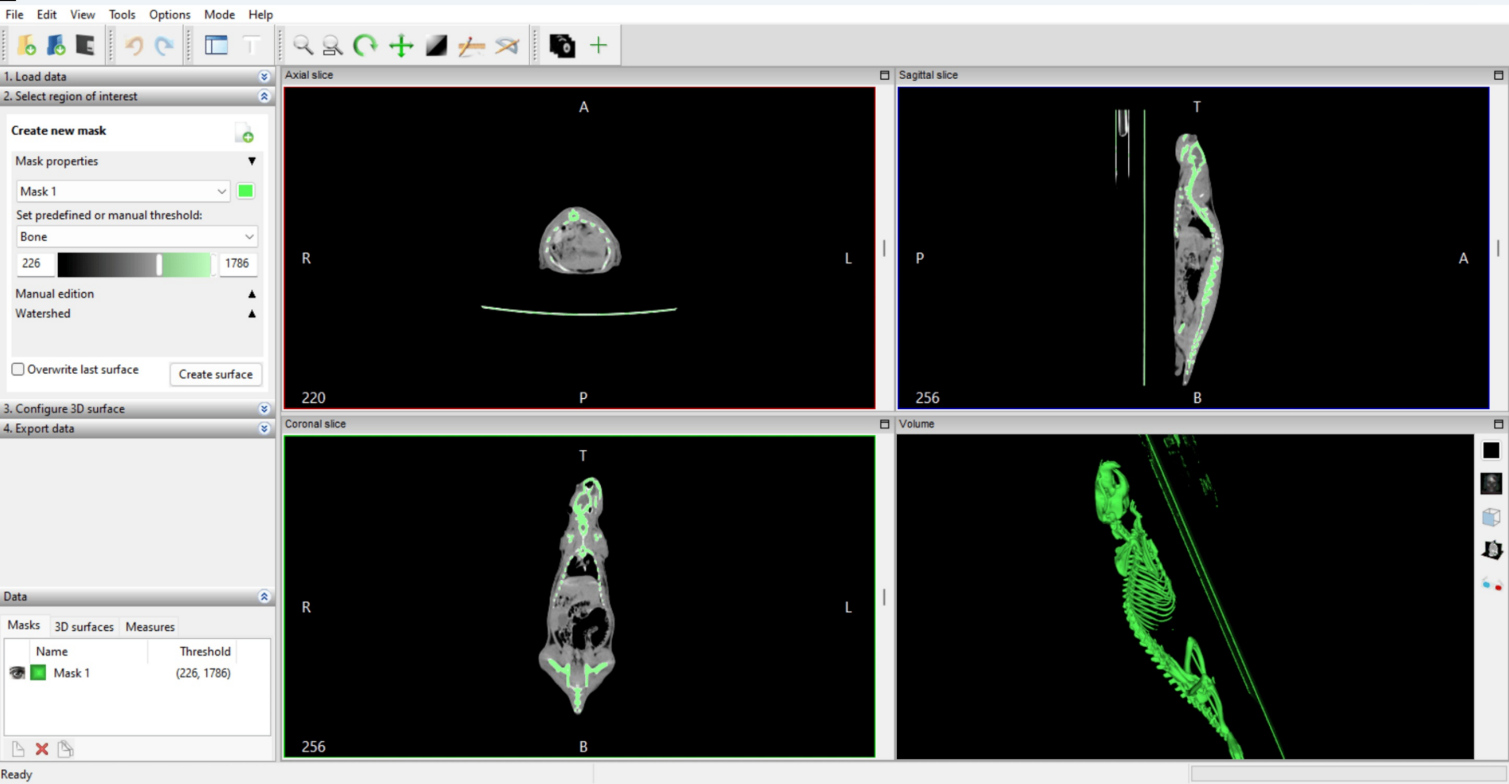
InVesaluis3D's program window with the extracted bone structure InVesaluis3D (Centro de Tecnologia da Informação Renato Archer, Campinas, Brazil)

The resulting 3D model, as shown in Figure 1, includes both the anatomical structure of the experimental animal and the representation of the object table (scanner bed) of the CT device. This additional detail highlights the context of the scan and provides a complete representation of the imaging setup.

After the initial reconstruction, the STL file was processed using Autodesk Meshmixer (Autodesk, Inc., San Francisco, CA). The main steps in this stage included the final segmentation of individual anatomical structures, including the os baculum (penile bone) and the os hyoideum (hyoid bone). Then smoothing and cleaning of the surface mesh to eliminate artifacts and improve the overall quality of the model. And finally preparation of the file for compatibility with 3D printing technologies, which involved checking the surface for connectivity and closing gaps or breaks in the mesh.

**Figure 2:**
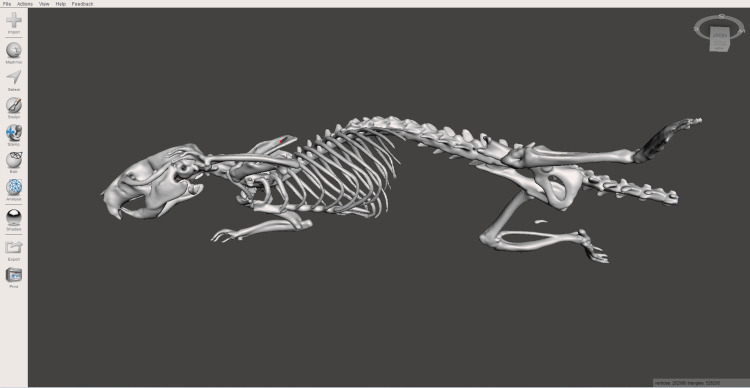
MeshMixer's program window with the finished bone structure

The resulting structure, shown in Figure 2, required minimal additional processing after the removal of the object table (scanner bed). The only adjustment necessary was the closure of an open surface in the caudal region of the model, which resulted from incomplete imaging of the tail. This limitation was due to a deliberate decision made to reduce the radiation dose absorbed by the experimental animal, following protocols established for imaging studies. The processed STL file is also available as an interactive model below in interactive Model 1.

**Video 1:** Rat bone structure

An FDM printer, the Bambu Lab X1 Carbon 3D Printer (Bambu Lab, Austin, TX), was used. This printer provides the high accuracy and precision required to reproduce intricate anatomical details. The material used for printing was PLA filament from 3Dline (3Dline, Yambol, Bulgaria), in a bone-coloured variant.

The selected printing settings included layer thickness of 0.2 mm, nozzle diameter of 0.4 mm, and tree-structured support, positioned to rest on the base of the print platform for the support material. Using these settings, the printing process required approximately 6.5 hours to complete. The printing setup ensured an optimal balance between accuracy and efficiency, maintaining high fidelity to the original anatomical structures without compromising on time constraints.

**Figure 3:**
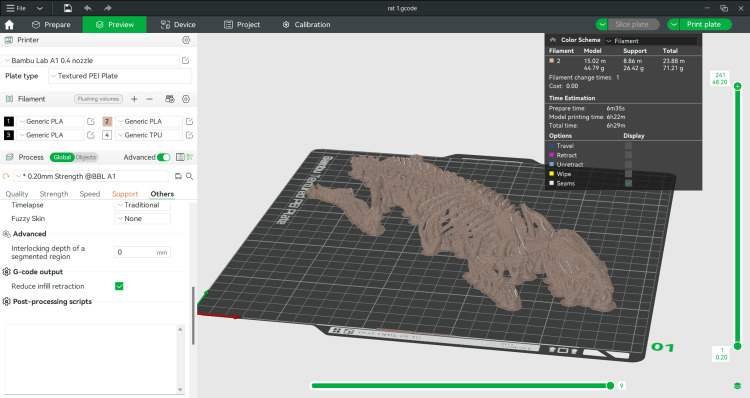
BambuStudio's program window with the finished bone structure BambuStudio (Bambu Lab, Austin, TX)

The program interface showing the sliced model is displayed in Figure 3. This visualization highlights the precise segmentation of the model and the placement of the support structures, providing users with a clear understanding of the pre-print preparation process. The use of tree-structured support minimized material waste while offering sufficient stability for complex overhangs, such as delicate bones and thin structures in the anatomical model.

**Figure 4:**
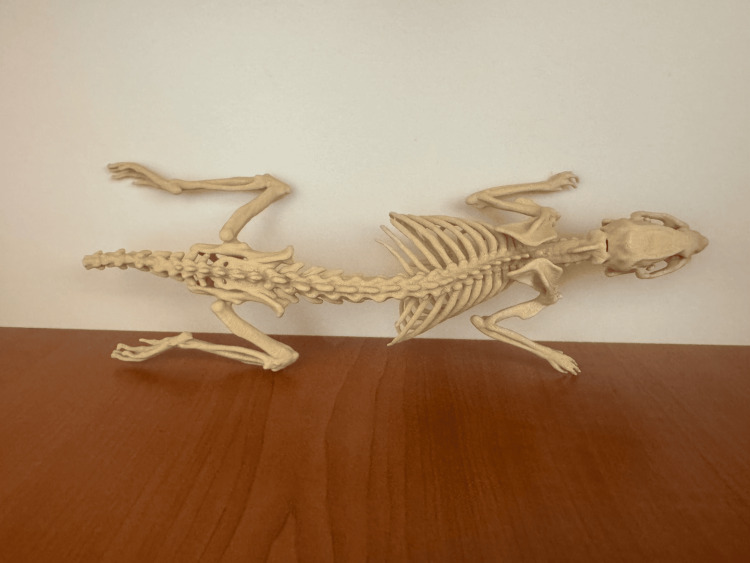
Printed educational model after post-processment

The choice of the bone-coloured PLA filament further enhances the model’s usability in educational and research environments, as it visually resembles natural skeletal structures, making it a valuable tool for anatomical studies and demonstrations. In Figure 4, the finalized 3D-printed model is displayed after the removal of the support structures. This step, performed manually, ensured the preservation of delicate anatomical details such as the os baculum (penile bone) and os hyoideum (hyoid bone), which are particularly susceptible to damage during post-processing. The removal of the tree-structured supports revealed the intricate features of the model with high precision, maintaining the anatomical fidelity achieved during the segmentation and printing stages. The cleaned model is ready for use in educational and research applications, providing a tangible, detailed representation of the Whistler rat’s anatomy.

The resulting 3D model is characterized by exceptional anatomical accuracy, including fine details often overlooked in traditional models. These details provide a more comprehensive representation of the anatomical structures, enhancing the model's value for educational and research purposes.

## Discussion

The development of the 3D model presented in this study demonstrates its significant potential as an educational and research tool. In addition to its precision, the model provides a valuable resource for medical and veterinary education, enabling students and professionals to explore complex anatomical features in a tangible and interactive format. This hands-on approach bridges the gap between theoretical knowledge and practical application, particularly in fields such as comparative anatomy, surgical training, and preclinical education. By offering an accurate and detailed representation of the subject's anatomy, the model supports deeper understanding and skill development in a range of disciplines. Preliminary user feedback and evaluations from educators or students can reinforce the model's educational utility. Similar models in the literature demonstrate improved learning outcomes and user satisfaction, as noted in recent investigations combining 3D technologies with AI in an educational context [10]. To ensure the accuracy of the 3D model, future studies should incorporate validation techniques, such as comparing reconstructed models with anatomical specimens using metrics like the Dice similarity coefficient or Hausdorff distance. Similar approaches in 3D printing validation, as discussed by [11], could highlight the importance of reproducibility in model creation.

The model's utility extends beyond education into research applications. Its high anatomical fidelity allows scientists to simulate experimental procedures, test hypotheses, and develop innovative methodologies [12]. This makes it particularly suitable for applications such as surgical planning, device testing, and biomechanical studies, where precision and reliability are of paramount importance. The inclusion of fine anatomical details, such as the os baculum (penile bone) and os hyoideum (hyoid bone), enhances the model's value, as these structures are often overlooked in traditional or generic anatomical models.

The optimization process applied to the STL file ensured its compatibility with common 3D printing technologies while maintaining a balance between anatomical accuracy and practical usability. This refinement included removing artefacts, optimizing geometry, and ensuring the structural integrity of the mesh. The resulting interactive model provides users with the ability to explore the reconstructed anatomy in detail, offering opportunities for customization, further analysis, or replication. By making this model openly accessible, the study fosters collaboration within the scientific community, encouraging broader adoption of 3D printing technologies in medical and veterinary practices. A similar approach to leveraging 3D scanning for detailed anatomical analysis has demonstrated the influence of positional changes on soft tissue landmarks, highlighting the importance of accuracy in model creation and validation [13]. This emphasizes the significance of ensuring anatomical fidelity and reproducibility, particularly when applying such models in diverse clinical and research contexts.

Despite its strengths, the model is not without limitations. Notably, the caudal structure (tail) is incomplete due to the deliberate restriction of the scanned area. This decision was made to adhere to protocols aimed at reducing the radiation dose absorbed by the experimental animal during the imaging process [14]. While this limitation reflects the ethical considerations inherent in preclinical studies, it also underscores the challenges of balancing imaging quality with safety concerns. The exclusion of the caudal region (tail) limits the model's applications in biomechanical studies or locomotion analysis. Alternative imaging methods with lower radiation exposure, such as microCT or dual-energy CT, could address this limitation, as suggested by [15]. Future studies may address such limitations by exploring alternative imaging strategies or techniques to minimize dose exposure while capturing complete anatomical features.

The workflow also highlights the practical considerations involved in developing accurate 3D models from imaging data. Factors such as the choice of imaging parameters, segmentation techniques, and post-processing methods all influence the final output. By integrating high-resolution imaging, meticulous segmentation, and advanced modelling techniques, the methodology delivers a detailed and reliable representation of the subject's anatomy. The chosen Hounsfield Unit (HU) range (226-1785) aligns with established practices for bone segmentation in CT imaging. Studies such as the one [15] demonstrate the need to justify HU thresholds by referencing tissue density ranges to ensure anatomical fidelity​

In conclusion, this study showcases how 3D printing technology can bridge gaps in education and research, providing detailed and accessible tools for a wide range of applications. The ethical and practical considerations addressed in this workflow further demonstrate the importance of thoughtful design and implementation in developing resources that are both impactful and responsible.

## Conclusions

The novelty of this analysis lies in its development of an accessible, customizable 3D anatomical model derived from experimental animal CT scan, addressing the limitations of traditional cadaveric and synthetic models. The study provides a practical solution for educational and research purposes by enabling open access to this resource, fostering broader usability and collaboration across disciplines.

This contribution has significant implications for the scientific and medical fields, offering a cost-effective and ethically viable alternative for anatomical studies. We believe that the approach supports enhanced adaptability to diverse educational needs, bridging the gap between theoretical learning and hands-on anatomical exploration.
